# Gendered Antecedents and Consequences of Young Women’s Suicidal Acts in Sri Lanka

**DOI:** 10.3390/ijerph20042885

**Published:** 2023-02-07

**Authors:** Jeanne Marecek, Chandanie Senadheera

**Affiliations:** 1 Department of Psychology, Swarthmore College, Swarthmore, PA 19081, USA; 2 Department of Psychiatry, Faculty of Medicine, University of Ruhuna, Galle, Sri Lanka

**Keywords:** suicidal acts, Sri Lanka, adolescent girls, gender norms, sexual respectability

## Abstract

In the late 1990s, Sri Lanka had a record rate of suicide deaths. Since then, deaths have decreased dramatically due to the restriction of lethal agrochemicals. The number of nonfatal suicidal acts, however, remains extraordinarily high. A disproportionate number of these cases are adolescents and young adults—mainly girls and young women. This paper offers a close look at adolescent girls in rural Sri Lanka who had engaged in nonfatal suicidal acts. We carried out interviews with daughters and mothers while the girls were receiving medical care following a suicidal act. Drawing from these interviews, we describe the circumstances leading to girls’ suicidal acts, the responses and moral judgments made by adult family members, and the reputational and social consequences of these acts. Few girls intended to die; none had previously undertaken a suicidal act, and none gave evidence of “mental illness”. In many cases, girls’ suicidal acts were triggered by acute family conflicts, often concerning situations that were seen to compromise the girl’s sexual respectability and the honor of her family.

## 1. Introduction

Every culture has a canonical narrative of suicide. Such narratives constitute a shared understanding of why people take their own lives. The narrative serves as a template that organizes suicidal acts undertaken by members of that culture [1]. It gives meaning to such acts as well as a moral accounting of them. Such narratives also shape the ways in which others relate to individuals who have engaged in a suicidal act. In Western high-income countries, the canonical narrative of suicide is provided by biomedical psychiatry. Suicidal behavior is said to be caused by a mental disorder, especially depression. Indeed, the fifth edition of the Diagnostic and Statistical Manual of Mental Disorders [2], as well as its subsequent updates, proposed a new diagnostic category, Suicidal Behavior Disorder [3]. However, although the claim that suicide is caused by a mental disorder is often stated as if it were a universal truth, research carried out by medical anthropologists and cultural psychologists reveals many other canonical narratives (e.g., [1,4,5,6,7]). 

This article concerns a cultural setting in which the canonical narrative of suicide does not link it to clinical depression or to any other mental disorder. This setting is Sri Lanka, a small island nation off the southeastern coast of India. There, the everyday understanding of suicidal acts is that those acts are driven by anger, by the desire to cast blame and shame on another person, or by a wish to “scare” others into doing one’s bidding. Such acts (or threats of such acts) are quite common among both women and men, and across the age span from mid-adolescence to old age. Neither psychiatrists, medical personnel, family members, nor individuals who have engaged in suicidal acts regard such acts as symptomatic of mental disorders. Following a suicidal act, individuals are typically admitted to medical wards; their physical symptoms are treated, and they are discharged. Especially if they are young, they may be deemed as “foolish”, “hotheaded”, or “uneducated”—bad but not mad. 

Some 25 years ago, Sri Lanka recorded 47 suicide deaths per 100,000, a death rate that was entered in the Guinness Book of World Records [8]. Most of these deaths resulted from the ingestion of agrochemicals, such as pesticides and weedicides, or poisonous plants, such as *kaneru* (yellow oleander). Following a sustained campaign to restrict the availability of lethal agrochemicals, the incidence of fatalities diminished dramatically [9]—a signal victory for the public health sector. However, the number of nonfatal suicidal acts did not decline; indeed, it may have increased [9]. Such cases are not tabulated in the national health statistics, nor consistently entered in hospital records. However, a door-to-door survey of households in the North Central province of Sri Lanka, a largely agricultural area, reported a rate of nonfatal self-harm of 560/100,000 [10]. 

It appears that adolescents and young adults constitute a large proportion of those who engage in nonfatal suicidal acts. Eighty-five percent of the cases reported in Pearson et al.’s household survey [10] involved adolescents and young adults. In the Teaching Hospital Karapitiya, where our studies were conducted, the medical records across several years showed that roughly 400 adolescents who had engaged in suicidal acts were admitted every year. In each year, girls outnumbered boys by roughly three to one [11]. Nearly all the adolescents (roughly 97%) survived. Some of the cases were not medically inconsequential, however. Some patients had swallowed substances such as bleach, rat poison, toilet cleaner, and kerosene oil, or they had ingested overdoses of various medications. 

In Sri Lanka, many suicidal acts are not undertaken with an intention to die. In fact, many suicidal acts are organized in ways that make death very unlikely to occur. Therefore, the terms “attempted suicide” and “suicide attempt”, which carry the meaning that the actor wished to die, are misleading. These terms—which are ubiquitous in research studies, in popular journalism, and in public information and education programs—erase the full range of motives and experiences of Sri Lankan individuals who engage in suicidal acts. Only a few of the girls who took part in our studies, for example, described themselves as intending to end their lives. Rather, their acts were meant as communications to others (and sometimes also about others). Tom Widger, a medical anthropologist who worked in the western coastal area of Sri Lanka, noted a similar pattern [12]. Drawing on his observations, Widger coined the term suicide-like act, which he defined as an act that was intended to put the idea of death into someone else’s mind. In what follows, we use the terms suicidal act, suicidal behavior, and suicide-like act interchangeably. We avoid altogether the terms suicide attempt and attempted suicide. 

Our goal was to study the meanings given to adolescent girls’ suicidal acts in the cultural and social contexts in which they were situated. Drawing from a larger study of young people who were admitted to medical wards following a suicidal act, we selected for this analysis the subset of participants who were adolescent girls. The analysis drew on interviews with these girls and with their mothers. 

We examined girls’ accounts of the circumstances and experiences that prompted them to engage in suicide-like acts, looking for common themes. We also examined the ways that mothers viewed their daughters’ suicidal acts. In mothers’ eyes, what were the reasons and causes behind their daughters’ suicidal acts? What moral judgments did mothers make about girls’ actions and the actions of others who were involved? We also took note of mothers’ and daughters’ expectations and fears regarding the familial and societal consequences of a girl’s suicide-like act. We scrutinized the strategies that mothers devised or contemplated in order to reduce the social harms and status injuries that might result from public knowledge of a girl’s suicidal act. 

### Norms of Femininity and Girlhood in Rural Sri Lanka 

Our knowledge interests centered on normative ideals of femininity, as well as the gendered and generational family relations that shaped girls’ actions and others’ responses. Here we provide a brief description of relevant aspects of feminine propriety and feminine ideals. We first discuss *læjja-baya* and the norms governing proper feminine demeanor [13,14]. Then we discuss the salience of marriage in young women’s lives and the importance for families of negotiating a “good” marriage for their daughters. 

The literal translation of *læjja-baya* is shame-fear—that is, a strong fear of being shamed. In Sri Lanka (and elsewhere in parts of South Asia), *læjja-baya* is a valued attribute, a quality that parents seek to inculcate in their offspring [14,15]. Beyond the fear of being shamed or criticized, the cultural ideal of *læjja-baya* also prescribes reticence, emotional restraint, and modesty, all of which are seen as key elements of proper public demeanor, especially for women. Further, girls and women ought to avoid actions that draw others’ attention to themselves. For instance, overt displays of emotion—whether anger, sadness, loud laughter, or physical gestures of affection—are frowned upon; such displays draw attention to the self. Such prescriptive norms regarding emotional restraint are considerably more stringent for women and girls than for men and boys. Keeping one’s emotions tightly in check is fundamental to proper female propriety and to a woman’s respectability. De Alwis [16] has traced the emergence of this ideal of feminine comportment to the confluence of traditional Buddhist dogma regarding emotional control and the Victorian ideals of womanly virtue brought to Sri Lanka by its British colonizers. 

We next consider the conventional meanings and practices of marriage in Sri Lanka. Although some members of the English-speaking urban elite have adopted the mores and practices typical of Western high-income societies, marriage (specifically, heterosexual marriage) remains the presumptive life status for adults in Sri Lanka. Indeed, getting marriage confers full adult status on a person. Most people expect and desire to be married and to have children. For the most part, being married, and having and raising children is a primary source of both personal identity and life satisfaction [17]. This is especially true for women. Moreover, marriage is more than the fulfillment of personal desire and romantic attraction; furthermore, having children is not solely a matter of personal preference. Both marriage and family formation are kin obligations—the obligation to carry the family lineage into the future. By longstanding tradition, parents bear responsibility for locating suitable marriage partners for their adult children and arranging for a marriage. By and large, young adults do not experience such parental involvement as intrusive or as an infringement on their autonomy; rather, it is a fulfillment of a pivotal family obligation [18]. 

For a young woman, marriage to a partner with good economic prospects or who comes from a prosperous family affords a means of upward economic mobility and higher social status for herself and her family. However, in order to strike a good match, a girl’s reputation must be unsullied; it cannot be compromised by rumors of sexual improprieties, romantic entanglements, or disrespectable behavior. Nor can her family’s background be tarnished by scandal. As we will see below, issues of reputational harm and scandal often came into play in the circumstances leading up to girls’ suicide-like acts and to families’ efforts to mitigate the consequences of these acts.

## 2. Method

The work we report here was carried out in the Teaching Hospital Karapitiya (hereinafter THK), located near the town of Galle in the southwest coastal belt of Sri Lanka. THK is the teaching hospital for the Ruhuna Medical Faculty and the only tertiary-care government hospital in the south of Sri Lanka. It is the third largest hospital in country. Because of its human resources and facilities, it is the referral center for the entire Southern province of Sri Lanka, serving mainly rural agricultural communities. 

The analyses we report here focus on a subset of interviews drawn from a larger project concerned with young people’s deliberate self-harm. The Institutional Review Board of the Faculty of Medicine of the University of Ruhuna reviewed the detailed protocol for the larger project and formally approved the project on 27 July 2007.

Chandanie Senadheera, a member of the Psychiatry Unit of THK and of the Ruhuna Medical Faculty teaching staff, was responsible for recruiting and interviewing participants for the study. Dr. Senadheera (hereinafter CS), who is a registered clinical psychologist, is the only psychologist in the hospital and on the Medical Faculty. 

### 2.1. Inclusion Criteria and Recruitment Procedure

The criteria for inclusion in the larger study were as follows: (1) the individual was admitted to the medical ward of the THK for inpatient treatment following an episode of self-harm; (2) the individual was between the ages of 15 and 18; (3) the individual’s medical condition had stabilized; (4) the individual agreed to be interviewed; and (5) a parent or an adult relative consented to the child’s participation. With the adolescent’s permission, CS invited their parent to be interviewed as well. 

With the assistance of the staff on the medical ward, CS monitored admissions to the ward. She met adolescents who had been hospitalized following a suicidal act after their medical condition had stabilized and invited them to take part in the study. Every patient admitted for a suicidal act who met the age criterion was invited to join the study. No one declined. 

### 2.2. Characteristics of the Study Group

In this paper, our focus is on the girls who were part of the larger project. We selected for analysis all the girls between the ages of 15 and 18 (N = 22). Thirteen of the girls were 15 or 16 and nine were 17 or 18. All but one came from Sinhala families, and all were at least nominally Buddhist. Nearly all their families lived in rural areas, as do 80% of Sri Lankans. Farm work and fishing were common means of livelihood. This means that household incomes varied widely and unpredictably in accordance with the season and the weather. Some fathers were in the military and others worked as migrant laborers in the Gulf States. Most mothers were not in the paid labor force, though some mothers worked as casual laborers on tea plantations or as sewing machine operators in garment factories. Most families lived in one-story houses with two or three rooms; most houses had electricity, but few had piped-in water. No family owned an automobile. 

All the girls had at least one sibling; most had two or more. None of the parents were divorced or formally separated, although a few fathers were deceased. As is often true in Sri Lanka, many girls had members of their extended family living nearby or even in the same household. All but two of the girls were in school (in one case, a girl had recently been forced to leave school to take a job because her father had unexpectedly died). 

Girls were asked for permission to interview their mothers as well. Every girl agreed to this. In Sri Lanka, female family members are expected to provide clean clothing, bed linens, and meals for hospitalized family members; moreover, it is customary for mothers to stay overnight with their daughters. Therefore, mothers were readily available both to be asked to consent to their daughters’ participation in the study and to be interviewed. 

### 2.3. Interview Procedures

Interviews were conducted in a private office away from the ward. All the interviews were conducted in Sinhala. Prior to the actual interview, the formal process of obtaining informed consent took place. CS introduced herself and explained that she wanted to learn more about the circumstances that led young people to engage in self-harm. She informed potential participants that their participation was voluntary; that they could choose not to answer any items if they wished; that they could withdraw from the interview at any time; and that the contents of the interview would remain anonymous and confidential. In addition to the oral description of the study, every participant was given a paper summarizing this information. Mothers and daughters independently provided written consent. 

### 2.4. Interview Format and Content

Daughters and their mothers were interviewed separately. Both daughters and mothers were told that nothing they said would be disclosed to the other party. The interviews were semi-structured. In the first segment of the interview, the questions/items were mainly open-ended. The goal was to gather rich talk that detailed the events leading up to the act of self-harm; the circumstances of the act itself; the thoughts and feelings the girl had when she decided to undertake a suicide-like act; and what had happened subsequently. CS opened the interview by asking simply “What happened?”—a question that implicitly asks for a narrative. As girls told their stories, CS used prompts and probes to help girls amplify their accounts. (E.g.: For how long had [the problem] existed? Do you remember what you were thinking about? How were you feeling? For how long did you think? Was anyone near you? Did anyone else know that you intended to harm yourself? Was there anything else you could have done? What did you think would happen? How did [your family] react when they realized what you had done? How/when did you get to the hospital? How has it been in the hospital?). The second segment of the interview was more structured. Girls were asked about their expectations of what would take place when they left the hospital (e.g., How do you think others—parents, other family members, neighbors, school friends—will treat you? Do you expect any changes in your life or in your relationships with others?). Girls were also asked if they knew others who had engaged in suicidal acts. Most said they did. They were asked to give a brief recounting of each of those acts and the aftermath. The mothers’ interviews covered similar topics as the daughters’ interviews, with the questions worded similarly. In many cases, mothers were cognizant of the angry responses and inflammatory suspicions that the girl’s suicidal act had engendered in the girl’s father and elder brothers. Mothers were also very attuned to the reputational harm that would follow if a girl’s suicidal act were publicly known. 

Most interviews lasted between 45 min and an hour. They were audio-recorded, transcribed, and then translated into English. 

### 2.5. Data Analysis

Both authors read the transcripts several times, working with both the original Sinhala and the English translation. In these readings, they made notes on “repeating ideas”, that is, ideas expressed by many of the participants [19]. We sought to understand the culture-specific meanings and moralities which daughters and mothers drew on to make sense of the girl’s suicide-like act. We also sought to identify common aspects of the circumstances that girls said had provoked their suicidal acts. Furthermore, we took account of the consequences—feared and actual—mentioned by girls and their mothers. Of course, we had no way to ascertain whether or not daughters’ or mothers’ accounts were fully accurate or fully truthful; our goal, however, was not to ferret out “the truth”. 

In what follows, all names are pseudonyms. Other possible identifying information, such as the names of villages or particular temples, have been changed or removed.

## 3. Findings

### 3.1. Overview

The descriptions that daughters and mothers gave of the girls’ suicidal acts included several features that set these acts apart from both completed suicides or attempted suicides. Most girls described their actions as largely unpremeditated. Sixty-five percent of them reported that they contemplated their suicidal act for 30 min or less. Only two girls reported contemplating the suicide-like act for as long as one day. In nearly every case, the girl’s home was the site where the suicidal act took place, and other family members were at home at the time. For the most part, the girls made minimal efforts (if any) to conceal what they had done. Some left empty pill bottles or empty cards of Paracetamol tablets in plain sight. Some had enlisted a little sister or brother to purchase cards of Paracetamol tablets at the neighborhood shop. Many girls very soon disclosed to their mother what they had done. In a few cases, such as the two that follow, girls dramatically enacted the self-harming act in others’ presence. 

Vimala (age 15) argued with her mother about whether or not she had “talked to” a boy on the bus ride home from school. Although Vimala said her mother’s accusation was false, her mother refused to believe her. “My head got hot”, Vimala said, “and it felt like it would explode”. She went to the back of the house, doused herself with kerosene oil, and, with a box of matches in hand, returned to face her mother. 

Sunila (age 16) realized that the neighborhood gossips had observed her alighting from the bus with a boy behind her. Knowing that she was in for a severe scolding from her home people, she tore through the house, screaming, “I’ll kill myself, I’ll kill myself”. In the kitchen area outside the back of the house, she quickly gulped some kerosene oil. 

In our reading of the circumstances of girls’ suicide-like acts, we see little evidence that the girls intended to die, or that they had a mental disorder, such as depression or borderline personality disorder (as a reviewer suggested). In no case had a girl previously engaged in a suicidal act. Moreover, in no case did the girl or her mother report that the girl had had previous psychiatric or psychological difficulties, a consultation with a mental health professional, or any form of psychological or psychiatric treatment. Indeed, many girls spontaneously reported that they had not intended their suicide-like act to end in death, but rather to force a change in someone else’s behavior. Furthermore, CS, the interviewer, is a registered clinical psychologist attached to the hospital’s psychiatry department. Had she observed signs or symptoms of a psychological disorder, she was authorized to refer a participant for a psychiatric evaluation. 

### 3.2. Antecedents of Girls’ Suicidal Acts

As has been consistently reported for over 20 years, suicidal acts (whether fatal or not) nearly always take place in the midst of interpersonal conflicts, usually involving family members [20]. This was also true for the girls who took part in this study. Their accounts of their suicide-like acts reveal that those acts were deeply embedded in girls’ social fields. Their stories offer a window into both ongoing relations with the family and the shared norms and values of the interpretive communities in which those families lived.

The stories vary widely in their specifics, but some storylines occurred over and over. In what follows, we describe the two most prominent ones, both of which involve threats to girls’ sexual respectability. We include excerpts from the interviews to illustrate these themes. 

#### 3.2.1. Suicidal Acts in Response to Sexual Accusations

Accusations of a romantic liaison (a “connection” with a boy) or sexual misbehavior figured in many of the stories. Like Vimala and Sunila, whom we described earlier, many girls gave accounts in which accusations of sexual misbehavior triggered the suicide-like act. Anu is one example. 

Anu (age 15) was the only girl in her family. She had five brothers. On the way home from school, she said, “A boy came after me and spoke to me. A relation of ours saw it. I got afraid because I knew he would tell my brothers. I rushed home and swallowed ten tablets of Panadol [the local brand of Paracetamol] […] My brother would have scolded me, but he won’t scold me now”.

Accusations by senior members of the family often involved fierce confrontations and harsh scolding. Not infrequently, girls’ stories told of physical assaults by their parents or elder brothers. In their interviews, mothers often unapologetically corroborated their daughter’s story. As the stories told by Mallika and Chalini portray, girls’ suicidal acts took place in response to these family altercations: 

Mallika (age 17) was to meet her mother at the bus stand in a neighboring town and travel home with her. They missed each other, however, and Mallika returned home alone. Her mother arrived home “in a great fury”. She surmised that Mallika had surreptitiously met a boy. She physically “assaulted” her daughter, snatched her cell phone, “dashed it on the ground and crumpled it”. Thirty minutes later, Mallika swallowed 30 Paracetamol tablets. Mallika said, “I was sad about what my mother did and I didn’t think of anything”. Reflecting on things some days later, Mallika amplified her story: “Because mother scolded me, I was angry and so I drank Panadol [a common brand of Paracetamol]. 

Chalini, (age 18) ingested a mixture of kerosene oil and crushed mosquito coils (i.e., coils soaked in mosquito repellent, which are burned in order to repel mosquitoes). Chalini had been forced to leave school to take up a job to support her family after her father’s unexpected death. A girlfriend found her a job in a garment factory. The factory was some distance from Chalini’s home, so she arranged to board with the girlfriend’s family. Within a few weeks starting the job, however, Chalini’s younger brother accused her of having an affair with a boy in the household. Her older brother forcibly dragged her back home. When Chalini disputed the accusation, both brothers, she said, “physically assaulted” her. In response, she swallowed the poison she had concocted. In hospital, Chalini said, “I felt sad and I drank poison”.

Other girls, such as Lakshmi, were subjected to taunts by their peers about (alleged) sexual misbehavior. Lakshmi was shamed by what she understood to be a schoolmate’s oblique reference to an out-of-wedlock pregnancy. In Lakshmi’s eyes, the taunt brought down shame on her and it dishonored her family as well. 

Lakshmi (age 16) had returned to school after several days’ absence. A girl in her class slyly asked her if she had “gotten married” while she was away. Lakshmi interpreted this remark as implying she had gotten pregnant. She felt sad, she said, and she thought about how sad the girl’s remark would make her mother. She bought a card of 10 Paracetamol tablets on the way home from school. When she got home, she swallowed them all. She found another card of Paracetamol tablets at home and swallowed those as well. Then she told her mother what she had done. 

To an outsider, the norms of feminine propriety to which unmarried female adolescents must conform seem inordinately stringent. Moreover, in a time when rural families are pushing daughters toward higher education and toward work in the paid labor force, such norms seem unsustainable. What is also striking is the array of community members who take it on themselves to surveil a girl’s actions—neighbors who tattle on the basis of slender evidence; peers who taunt her; and brothers (elder and younger) who have license to police her actions and beat her for what they regard as infractions. Among the families in this study, both generational and gendered hierarchies of power seem firmly held in place. 

#### 3.2.2. Suicidal Acts as Strategic Efforts to Force a Change in Others 

For some girls, a suicide-like act was a desperate attempt to intervene in their father’s excessive drinking. In rural Sri Lanka, habitual alcohol misuse is widespread among men [21,22]. Men’s drunkenness spawns chronic health problems, spousal violence, economic problems, and strife within families. It often leads to mayhem, street brawls, and trouble with the law. Habitual drunkenness and uncouth behavior by male members bring disgrace to the family and sully the family’s good name. As Ramani and Ayesha well understood, a father who was known as a drunkard diminished the prospects of a good marriage for his daughters. Distressed by the implications of their fathers’ behavior, they resorted to suicidal acts as a means to impress on them the need for change. 

Ramani, age 17, had a father who was a heavy drinker. Often he was found lying on the road, unconscious and unclothed. Ramani reported that she frequently lay awake at night, thinking about “all the things he was doing…” Ramani said, “When they behave like that, there will be no future for grown-up girls”. [This statement alludes to the likelihood that her father’s continual public intoxication would negatively affect the family’s chances of arranging a good marriage for herself and her sisters.] Ramani swallowed several Paracetamol tablets. 

Ayesha, age 18, swallowed 36 Paracetamol tablets because she was distressed by the public display of her father’s drunken shouting, boisterous singing, and uncouth behavior. In the hospital, Ayesha told CS, “I got so wild. I got down the tablets through my younger sister [i.e., she sent her younger sister to purchase them at the local shop.] and took them”.… “I had in my mind that I will punish him and frighten him … not that I wanted to die. I wanted to somehow see that father would not drink thereafter. I decided to put a stop to it today itself. … It came to my mind that if I do a thing like this, he will not drink hereafter”. 

Ayesha’s mother concurred with Ayesha’s account, adding that Ayesha “felt ashamed when her father was shouting loudly … She tells father not to drink because all are girls in the family. She is ashamed of it”. 

Kanthi’s suicide-like act was also undertaken as a means to force another person to stop behavior that was detrimental to her. Kanthi, age 15, had received repeated marriage proposals from a 19-year-old cousin. With the agreement of her family, she had refused these proposals. She was too young to be legally married, and she needed to remain in school. Nor did she like the boy. To coerce her into agreeing to marry him, the boy took an overdose of sleeping pills. From the hospital, he asked his sister to take a letter to Kanthi. In the letter, he threatened to take a lethal dose of poison unless she agreed. “If anything happens to me”, the letter said, “my family will ruin your family. They will not be quiet”. Kanthi countered his threat by swallowing two packets of a highly toxic bleaching agent. In hospital, she told CS triumphantly, “Now they won’t harass my family anymore”.

### 3.3. Families’ Responses to Girls’ Suicidal Acts 

Thus far, we have focused mainly on girls’ accounts of their suicidal acts. In those accounts, girls positioned themselves as having temporarily experienced acute feelings of upset, sadness, or anger, telling stories of interpersonal situations that were difficult, painful, or unfair. In most cases, their mothers were not inclined to be sympathetic. Mothers expressed little interest in their daughters’ emotional distress. Mothers (along with many hospital nurses and doctors) repeatedly used the phrase “a foolish thing” to berate their daughter for what she had done. They interpreted the suicide-like act as an indication of a lack of proper self-control, of “stubbornness”, or of “disobedience”. Some daughters described their mother’s angry response to their suicidal act. For example, a 15-year-old described her mother this way: 

First she beat me. Then she threw me into a 3-wheeler [a three-wheeled taxi] and rushed me to the hospital. 

Some mothers viewed their daughter’s behavior as a willful lack of consideration for the welfare of other family members. A mother of a 17-year-old girl told CS: 

On the way to the hospital, I told her that if she happens to die, both mother and father will have to languish in jail. Your younger sister and younger brother will be on the road where they will have no one.

(This is, of course, an exaggeration. Parents are not jailed because of a child’s suicidal act, nor are young children left “on the road” by their families.) 

CS did not speak directly to fathers, few of whom visited the hospital. However, mothers often expressed worry regarding the extremity of the reactions of their husbands and older sons. Some mothers noted that their husbands were upset and shamed by their daughter’s hospitalization. However, other mothers described their husbands’ mood as angry. One mother recounted her husband’s response when she told him that their daughter had swallowed an overdose of Paracetamol:

Suddenly father jumped up, asked me the reason why, and shouted at me. Father has become furious. After she was brought to the hospital, he said that she can no longer live with him, having had such a thing in her mind. 

A number of mothers said that their husbands and elder sons intended to punish the girl when she returned from the hospital. One mother said:

I know she will be beaten when she gets home. They are in a fury, and I fear their rage.

Another mother said:

Father plans to beat the girl when she gets home. I have to find a way around that.

According to many mothers, fathers and elder brothers assumed that the girl’s suicidal act was proof that she had lost her virginity. The plans men entertained for rectifying this were quite extreme. For instance, some fathers and elder brothers, presuming that there had been sexual “connection”, were intent on locating the boy and then beating up him and his father. In one instance, a father and his elder son had armed themselves with knives and attacked the boy whom they believed had “defiled” the girl. The police had intervened, and a court case was pending. Other fathers were prepared to force their daughters into a marriage if the girl had been “spoiled”. It mattered little if the boy was a “bad character” or his family was “bad” or the girl was too young to be married legally. In many cases, mothers expressed strong reservations about the wisdom of their husband’s plans, and they said they were seeking ways to prevent him from putting them into effect. 

In sum, for girls’ parents, their daughter’s suicide-like act precipitated a crisis. In no case, however, was this seen as a mental health crisis. For mothers, the girl’s action was a display of lack of control over her emotions and of willfulness. It was evidence as well of her disregard for protecting the honor and “face” of the family. However, for fathers, the salient meaning of their daughter’s suicidal act was the (presumed) violation of her sexual purity; punitive measures were foremost on their mind. 

### 3.4. Suicide-like Behavior and a Spoiled Identity 

As mothers and daughters well knew, public knowledge of a girl’s suicidal act would damage her reputation and diminish the family’s status. Village gossipmongers would spread the word, and people would infer that some kind of sexual misbehavior or sexual defilement had been involved. The young woman would no longer be regarded as a “good girl”. Therefore, secrecy was a paramount concern for both mothers and daughters. A mother, for example, said:

It’s more important to keep it a secret if it’s a girl; we all know how people gossip.

Commenting about another girl’s suicidal act, a girl said: 

People say various things, and they say that she is not a good girl. 

Another girl said: 

I will not be able to face others, as the villagers will say this and that.

The mother said this:

Now also no one in the village knows about it, as it can be a problem for her. The villagers come out with other problems. [“Other problems” is likely a euphemism for sexual misbehavior.]

A mother reported that her family was determined to keep the daughter’s suicidal act a secret … because people are waiting to laugh at us. 

Mothers took a variety of steps to shroud a daughter’s suicidal act in secrecy. Some fabricated false diagnoses (e.g., a bout of gastritis; an allergic reaction to medication) to explain their daughters’ hospitalization. Others took precautions to hide their daughters away from hospital employees who were neighbors. Some entered fictitious addresses in the hospital records. Some mothers thought that they might send their daughter to live with distant relatives until the local rumormongers turned their attention elsewhere. Some mothers intended to confine their daughter at home. 

Mothers’ concerns about the potential damage of exposing a girl’s suicidal act were not unfounded. During follow-up interviews, some girls reported experiences of social exclusion. Some girls had been living in boarding houses or staying with relatives. In every such situation, the girl was not allowed to return. The owner of one boarding house told the girl’s mother: 

Her coming back here would create a problem for the other girls.

In some cases, the school principal refused to allow girls to return to school. Some girls were barred from private tuition classes they had been enrolled in. In at least one case, the parents of a girl’s best friend forbid their daughter from associating with her. In short, mothers’ concerns about the social and reputational harm that a girl’s suicide-like act would do seemed quite justified. Further, the various forms of social exclusion that girls experienced seemed to coalesce around a concern that the girl was no longer a “nice” (proper and morally upright) girl. Her presence among other girls would be a source of contamination. 

## 4. Conclusions

Every year, high numbers of girls in rural areas of Sri Lanka are admitted to hospital following their ingestion of household poisons or medicinal overdoses. Girls arrange these suicidal acts in ways that strongly suggest that the acts are not intended to cause death. Among the participants we studied, many of such suicidal acts sprung from clashes within families, often involving girls’ sexual respectability. Mothers regarded their daughter’s suicidal act as “foolish” and headstrong. It also signified the girl’s disregard for her family’s status. For young unmarried women, a suicide-like act automatically sparked rumors of sexual impropriety, jeopardizing both the girl’s honor and the family’s good name. 

The meanings, practices, and aftermath of girls’ suicidal acts offer a window into the gendered expectations, constraints, and demands placed on young unmarried women. We offer a brief description of boys’ suicidal acts to highlight disparities between the girls and boys. First, only about a third as many boys as girls were hospitalized for a suicidal act. Second, among the boys we interviewed, there were no instances in which an accusation of sexual impropriety played a role. Third, boys’ suicidal acts usually took place outside the home at school or among their friends. Sometimes the boys were involved in “suicide play” with their friends, for example, “daring” one another to drink poison [23]. Third, no boy spoke of having been “assaulted” by a parent or elder sibling when he disclosed his suicidal act, nor were there reports of a boy being barred from attending school or turned out of his place of residence for fear of “contaminating” other boys. The number of boys we were able to interview was too small to permit further analysis, however, even this cursory discussion suggests that, among adolescents at least, suicidal acts are highly gendered. 

In the rural setting of this study, girls’ suicide-like acts often arose in the context of family conflicts regarding feminine propriety and sexual respectability. At the same time, suicidal behavior seemed inevitably to arouse societal anxieties about sexual misbehavior or sexual violation. Why do the suicidal acts of girls seem to evoke scolding, punishment, and social exclusion when those of boys do not? Why do families regard concealment of a suicidal act as so crucial for girls? The reasons, we surmise, lie in the negative consequences that such acts may have for both girls and their families. In Sri Lanka, the canonical narrative of suicide attributes it to anger and a desire for vengeance. Such motives stand in sharp contrast to prescriptive norms for girls such as docility, obedience, and *læjja-baya.* Furthermore, girls’ suicidal acts—no matter the actual circumstances—seemed inevitably to give rise to speculations about sexual misconduct. As we noted earlier, a “good” marriage (that is, a marriage that betters a family’s economic and social status) depends on a girl’s good reputation. Public knowledge of a girl’s suicidal act may diminish her family’s chances for upward social and economic mobility. 

We note that social and economic changes wrought by modernization come into conflict with stringent standards of feminine modesty and propriety [24]. Consider Chalini, for instance, whom we described above. The straitened circumstances of Chalini’s family required her to leave school and take a job. However, just a few weeks after she began to work, someone floated a rumor about a sexual liaison, which impugned her (and her family’s) respectability. Even though Chalini denied the rumor, her brothers forced her to quit her job and return home. Contentious arguments between Chalini and her brothers led to physical fights, and ultimately to her suicidal act. More generally, the present-day circumstances of everyday life for many adolescents no longer permit parents to monitor their daughters’ movements and acquaintances. For example, many rural families arrange for their adolescent daughters to pursue schooling beyond what the village school provides. Consequently, many girls ride public buses to schools some distance from home. During these journeys, young people intermingle without parental surveillance. This is just one of many instances in which the degree of surveillance and control that rural parents had over earlier generations of adolescents is no longer possible.

We next consider possible applications of this work. We first note the acute lack of resources for mental health care. Although Sri Lanka is often touted for its free health care, behavioral health care has lagged far behind. Psychiatrists are concentrated in a few large cities; even there, mental health care is accessible mainly to the cosmopolitan elite. The national health system, which serves a population of 22 million, employs only three psychologists. The THK, the third largest tertiary care hospital in the country, serves a catchment area of roughly two and a half million people yet it employs only four psychiatrists and one psychologist. 

We remind readers that in Sri Lanka, the canonical narrative of suicide holds suicidal acts to be caused by anger and a desire for revenge, not by mental disorder. This narrative organizes suicidal acts, families’ responses to such acts, and the medical care given to suicidal individuals. In the THK, for example, people who have engaged in suicidal acts are admitted to a medical ward, where their physical injuries are treated. When the physical condition permits, the patient is discharged. In the unusual case where there is also evidence of a psychological disorder or a prior history of psychiatric hospitalization, a patient can be transferred to the psychiatric ward. 

Some readers may believe that suicidal individuals in Sri Lanka ought to be given the psychiatric diagnoses conventionally given to suicidal individuals in Western countries. We disagree. This, of course, is germane to longstanding debates about the transcultural validity of Western-centric diagnostic categories, a debate that we cannot rehearse here. We note simply that because of the unavailability of psychologically oriented treatments, a patient would derive little benefit from receiving a psychiatric diagnosis. In fact, receiving such a diagnosis likely would be detrimental. There is a lasting stigma associated with being labeled as being *pissu* (crazy) or as having a *manasika rogaya* (disease of the mind). Such labels incur a stigma for both patient and family. It is for this reason that many families shun services offered under the auspices of psychiatry. Instead, they might seek out the services offered by indigenous healers, including astrologers and palmists who offer diagnoses and prognoses, and ritual specialists who can provide curative charms and amulets. In addition, many people engage in spiritual practices such as *Bodhi puja* (ritual worship of the Buddha) or chanting *seth kavi* (auspicious verses). 

Our work and that of others underscores that suicidal acts are relational practices that accord with the prevailing social order and the cultural ethos. In rural Sri Lanka, suicide-like acts (and threats of same) are commonplace. “Suicide games” are an everyday element of childhood play [23]. Often, suicide-like acts (and threats of same) are the means by which persons lower in status (e.g., wives vis à vis their husbands; young adults vis à vis their parents) gain leverage over the other’s behavior [24]. For example, threatening suicide by feigning a gulp of insecticide is a commonplace way for a wife to try to curtail her drunken husband’s violence. The girls we studied told stories about neighbors, relatives, and household members who had engaged in suicidal acts. In their villages, such occurrences were not extraordinary. Moreover, the girls did not criticize those individuals. In fact, some girls viewed such suicidal acts as effective means to force others to change. One of us (JM), working with rural school counselors, noted a similar endorsement of the use of suicidal threats as a useful way to force behavior change [25]. Altering this generalized cultural endorsement of suicidal acts is a significant challenge for anyone interested in suicide prevention.

Finally, in our view, it is important to move away from the conventional focus on presumed deficiencies or pathologies of those who engage in suicidal acts. Ought we to prescribe “anger management” for a wife whose drunken husband threatens her with ax? Does an adolescent whose mother flies into a “towering rage”, beats her with a belt, and demolishes her cell phone need better “coping strategies?” Is the girl’s anger a symptom of Emotion Dysregulation Disorder? Further, is there any evidence that those who engage in suicidal acts lack “decision-making skills” or “self-esteem?” Whether intended or not, such person-centered attributions blame the victim. In place of speculations about individual deficits and pathologies, we urge attention to the cultural, structural, and relational conditions that set the stage for such suicidal acts. In rural Sri Lanka, for example, wife abuse is commonplace and widely condoned. Chronic drunkenness among men is an unremarkable fact of life, which carries few legal repercussions. Children and adolescents are subjected to shaming and harsh physical punishment both at home and in school. Rigidly enforced generational hierarchies give rise to strained relations among extended family members. Economic precarity fuels family conflicts. Ameliorating the structural and cultural conditions of rural life may be an effective means of curbing the incidence of suicidal acts. 

## Data Availability

In order to protect the privacy of the research participants, interview transcriptions cannot be made available.

## References

[1] Andriolo K. (1998). Gender and the cultural construction of good and bad suicides. Suicide Life-Threat. Behav..

[2] American Psychiatric Association, DSM-5 Task Force (2013). Diagnostic and Statistical Manual of Mental Disorders: DSM-5™.

[3] Fehling K.B., Selby E.A. (2021). Suicide in DSM-5: Current evidence for the proposed Suicide Behavior Disorder and other possible improvements. Front. Psychiatry.

[4] Canetto S.S. (2015). Suicidal behaviors among Muslim women: Patterns, pathways, meanings, and prevention. Crisis J. Crisis Interv. Suicide Prev..

[5] Lee S., Kleinman A., Perry E.J., Selden M. (2000). Suicide as resistance in Chinese society. Chinese Society: Change, Conflict, and Resistance.

[6] Marecek J., Senadheera C. (2012). “I drank it to put an end to me”: Sri Lankan girls narrate suicide and self-harm. Contrib. Indian Sociol..

[7] Staples J. (2012). The suicide niche: Accounting for self-harm in a South Indian leprosy colony. Contrib. Indian Sociol..

[8] Gunnell D., Eddleston M. (2003). Suicide by intentional ingestion of pesticides: A continuing tragedy in developing countries. Int. J. Epidemiol..

[9] De Silva V.A., Senanayake S.M., Dias P., Hanwella R. (2012). From pesticides to medicinal drugs: Time series analyses of methods of self-harm in Sri Lanka. Bull. World Health Organ..

[10] Pearson M., Metcalfe C., Jayamanne S., Gunnell D., Weerasinghe M., Pieris R., Priyadarshana C., Knipe D.W., Hawton K., Dawson A.H. (2017). Effectiveness of household lockable pesticide storage to reduce pesticide self-poisoning in rural Asia: A community-based, cluster-randomised controlled trial. Lancet.

[11] Senadheera C., Marecek J. (2018). Deliberate self-harm in adolescents in southern Sri Lanka: A hospital-based study. Galle Med. J..

[12] Widger T. (2015). Suicide in Sri Lanka: The Anthropology of an Epidemic.

[13] Abeyasekera A.L., Marecek J. (2019). Embodied shame and gendered demeanours in young women in Sri Lanka. Fem. Psychol..

[14] Obeyesekere G. (1984). Medusa’s Hair.

[15] Spencer J. (1990). A Sinhala Village in a Time of Trouble: Politics and Change in Rural Sri Lanka.

[16] De Alwis M., Jeganathan P., Ishmail Q. (1997). Gender, politics, and the “respectable lady”. Unmaking the Nation.

[17] Abeyasekera A.L. (2021). Making the Right Choice: Narratives of Marriage in Sri Lanka.

[18] Abeyasekera A.L. (2017). “Living for others”: Narrating agency in the context of failed marriages and singleness in urban Sri Lanka. Fem. Psychol..

[19] Marecek J., Magnusson E. (2015). Doing Interview-Based Research: A Learner’s Guide.

[20] De Silva H., Kasturiaratchi N., Seneviratne D., Senaratne D.C., Molagoda A., Ellawala N. (2000). Suicide in Sri Lanka: Points to ponder. Ceylon Med. J..

[21] Gamburd M.R. (2008). Breaking the Ashes: The Culture of Illicit Liquor in Sri Lanka.

[22] Wickramasinghe A. (1993). Changing family life: Case of men’s alcoholism in rural Sri Lanka. Int. J. Sociol. Fam..

[23] Widger T., Broz L., Münster D. (2015). Learning Suicide and the Limits of Agency: Children’s “Suicide Play” in Sri Lanka. Suicide and Agency: Anthropological Perspectives on Self-Destruction, Personhood, and Power.

[24] Abeyasekara A.L., Marecek J. (2021). Transnational feminisms and psychologies: Selves, suffering, and moral personhood in Sri Lanka. Women & Therapy.

[25] Marecek J., Johnson K., Cunningham L., Walker C.J. (2012). From data to action: A culturally sensitive intervention for adolescent self-harm in Sri Lanka. Community Psychology and the Economics of Mental Health: Global Perspectives.

